# Post-marketing surveillance of the safety and effectiveness of nivolumab for classic Hodgkin lymphoma in Japan

**DOI:** 10.1007/s12185-024-03734-y

**Published:** 2024-03-23

**Authors:** Akira Kawasaki, Kiyohiko Hatake, Itaru Matsumura, Koji Izutsu, Tomohiro Hoshino, Ayumi Akamatsu, Akito Kakuuchi, Kensei Tobinai

**Affiliations:** 1 Ono Pharmaceutical Co., Ltd, 1-5, Dosho-machi 2-chome, Chuo-ku, Osaka, 541-8526 Japan; 2 Department of Hematology, International University of Health and Welfare, Tokyo, Japan; 3 Department of Hematology and Rheumatology, Faculty of Medicine, Kindai University, Osaka, Japan; 4 Department of Hematology, National Cancer Center Hospital, Tokyo, Japan

**Keywords:** Classic Hodgkin lymphoma, Japan, Post-marketing surveillance, Safety

## Abstract

Nivolumab was approved for relapsed/refractory classic Hodgkin lymphoma (cHL) in Japan in 2016. After its approval, a prospective, non-interventional, observational post-marketing surveillance was initiated to evaluate the safety and effectiveness of nivolumab treatment for up to 12 months in patients with relapsed/refractory cHL. Of 304 registered patients, 288 were included in safety analyses and 282 in effectiveness analyses. There were 191 (66.3%) male patients, median age was 64.0 years, and 54 patients (18.8%) had performance status ≥ 2. Treatment-related adverse events (TRAEs) were reported in 183 (63.5%) patients, with grade 3–5 TRAEs in 86 (29.9%). The most common TRAEs were infusion reaction (14.6%), hepatic function abnormal (5.9%), interstitial lung disease (ILD) (5.6%), and hypothyroidism (5.2%). TRAEs of special interest in ≥ 5% of patients were infusion reaction (15.6%), hepatic failure/hepatic dysfunction/hepatitis/cholangitis sclerosing (13.2%), thyroid dysfunction (9.7%), and ILD (7.3%). In multivariable analyses, prior allogeneic hematopoietic stem cell transplantation was a risk factor for hepatic failure/hepatic dysfunction/hepatitis/cholangitis sclerosing, and prior thyroid gland disorders was a risk factor for thyroid dysfunction. The overall response rate was 61.7%. In conclusion, nivolumab showed a similar safety profile and comparable effectiveness to that reported in clinical trials for relapsed/refractory cHL (CheckMate 205, ONO-4538-15).

**Supplementary Information:**

The online version contains supplementary material available at 10.1007/s12185-024-03734-y.

## Introduction

Classic Hodgkin lymphoma (cHL) is a localized or disseminated malignant proliferation of B-cells that accounts for about 30% of all cases of malignant lymphoma in the United States of America and Europe and about 6% in Japan [1, 2]. Hodgkin and Reed–Sternberg (HRS) cells exist in the tumor microenvironment [3] and overexpress programmed death (PD) ligand-1 (PD-L1) and PD-L2, contributing to immune evasion [4]. Blocking this pathway is a promising treatment approach for cHL.

Nivolumab was the first humanized monoclonal antibody targeting human PD-1 [5] that restores the ability of cytotoxic T lymphocytes to eliminate tumor cells [6, 7]. Clinical trials conducted globally (CheckMate 205 [8, 9]) and in Japan (ONO-4538-15 [10, 11]) led to the approval of nivolumab for treating relapsed/refractory (r/r) cHL in December 2016 in Japan. Nivolumab was subsequently incorporated into the current National Comprehensive Cancer Network Guidelines [12] and the Japanese Society of Hematology guidelines for HL [13].

CheckMate 205 and ONO-4538-15 were limited by their sample sizes (80 and 17 patients, respectively) and stringent inclusion/exclusion criteria. Therefore, their results may not fully reflect the real-world setting, where many patients who would have been excluded from the clinical trials are treated with nivolumab.

Upon its approval in Japan, a post-marketing surveillance (PMS) was conducted to collect safety and effectiveness data for all patients treated with nivolumab for cHL in real-world settings at the request of the Ministry of Health, Labor and Welfare, Japan. We report the final results for all patients registered up to December 31, 2019, and treated with nivolumab for cHL.

## Materials and methods

This prospective, non-interventional, observational PMS evaluated the safety and effectiveness of nivolumab for 12 months after the first dose in patients with r/r cHL in Japan. This PMS conformed to Japanese Good Post-Marketing Study Practice regulations. Each participating hospital agreed to contracts with the study sponsor. Approval from the institutional review board or ethics committee and collection of written informed consent from the patients, although not mandatory, were obtained depending on the sites’ requirements. This PMS was registered on the Japan Registry of Clinical Trials (https://jrct.niph.go.jp/latest-detail/jRCT1080224013).

All patients with cHL intended to receive nivolumab after its approval date (December 2, 2016) were centrally registered. Although it was planned to continue registration until November 2022, we only collected case report forms for patients registered through to December 31, 2019, when the planned number of patients had been reached. We report the data for patients treated at 195 hospitals, which provided consent for data publication.

According to the approved label, nivolumab was intravenously administered at a dose of 3 mg/kg every 2 weeks or 240 mg/body every 2 weeks. Patients were monitored for 12 months after their first dose. Patients who discontinued nivolumab treatment within 12 months were also followed up, if possible, for 12 months after the first dose. The case report forms, which were completed by the physicians, included baseline demographic characteristics, such as age, sex, Eastern Cooperative Oncology Group performance status (ECOG PS), medical history, Ann Arbor staging classification, and prior treatment for cHL, and nivolumab administration status (number of doses and the reason for discontinuation).

The primary outcome was the incidence of treatment-related adverse events (TRAEs), which were classified by the sponsor according to system organ class/preferred term using the Japanese version of the Medical Dictionary for Regulatory Activities (MedDRA) version 24.0. The physicians graded the TRAEs using the National Cancer Institute Common Terminology Criteria for Adverse Events versions 4.0 or 5.0. TRAEs were evaluated in all patients and in subgroups of patients stratified by their baseline characteristics.

In accordance with the Risk Management Plan, the following adverse events (AEs) were included in the safety specification and defined as TRAEs of special interest in this PMS: interstitial lung disease (ILD), myasthenia gravis, myocarditis, myositis, rhabdomyolysis, colitis, enteritis, severe diarrhea, type 1 diabetes mellitus, hepatic failure, hepatic dysfunction, hepatitis, cholangitis sclerosing, thyroid dysfunction, pituitary disorder, neurological disorder, renal disorder, adrenal disorder, encephalitis, severe skin disorder, venous thromboembolism, infusion reaction, serious blood disorder, hemophagocytic syndrome, tuberculosis, and cardiac disorders such as atrial fibrillation, bradycardia, and ventricular extrasystoles.

At 6 and 12 months after the start of nivolumab treatment, or earlier if treatment was completed/discontinued, the best tumor response achieved in individual patients (categorized as complete response [CR], partial response [PR], stable disease [SD], progressive disease [PD], or not evaluable) was assessed by the treating physician, according to the 2007 Report of an International Workshop to Standardize Response Criteria for Non-Hodgkin’s Lymphoma [14]. The overall response rate (ORR) was calculated as the proportion of patients with a best response of CR or PR.

The safety analysis set comprised all registered patients excluding those treated at facilities that did not grant permission for the analysis, patients who did not receive nivolumab, and duplicated patients. The effectiveness analysis set comprised all patients in the safety analysis population, excluding patients concomitantly using other anticancer agents, patients without prior chemotherapy, and patients with an unknown dose/administration period.

The frequencies of TRAEs were compared among subgroups of patients using Fisher’s exact test, Wilcoxon’s rank-sum test, or the χ^2^ test, as appropriate, with cross-tabulations with other items for factors showing significant differences among subgroups. To identify patient characteristics that may be associated with the risk of thyroid dysfunction, hepatic dysfunction (hepatic failure/hepatic dysfunction/hepatitis/cholangitis sclerosing), or ILD, we performed competing risk analyses using the Fine and Gray proportional subdistribution hazards model.

SAS statistical software (SAS Institute Japan Ltd.) version 9.4 TS1M4 was used for all statistical analyses.

## Results

### Patient characteristics

**Fig. 1:**
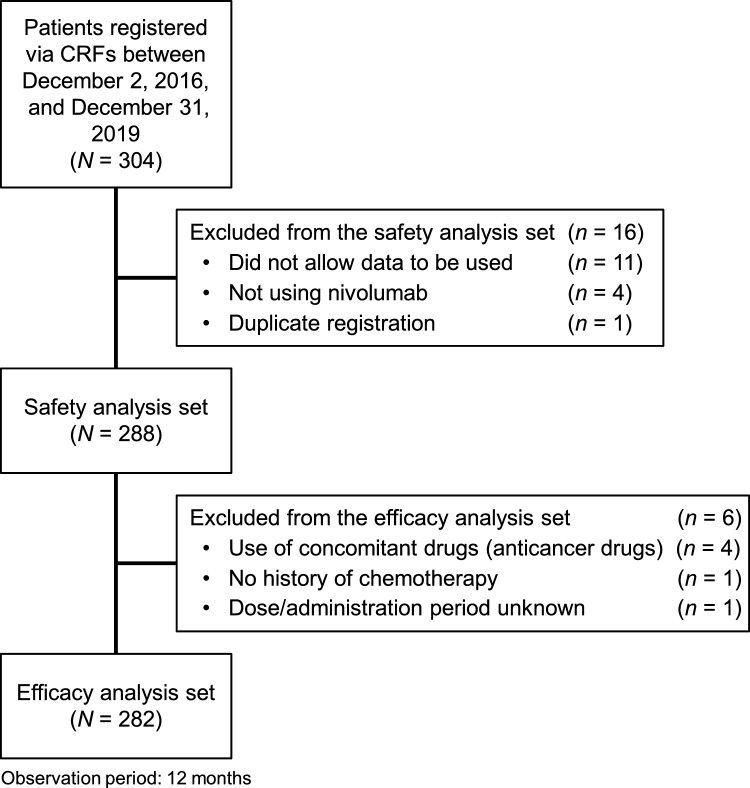
Patient disposition. *CRF* case report form. Patients were registered between December 2, 2016, and December 31, 2019

Of 304 patients with cHL registered between December 2, 2016 and December 31, 2019 (Fig. 1), 288 were included in the safety analysis set, and 282 were included in the effectiveness analysis set. The median age of patients in the safety analysis set was 64.0 years and 68 patients (23.6%) were ≥ 75 years old. There were 191 males (66.3%), and 54 patients (18.8%) had an ECOG PS ≥ 2. Twenty-three patients (8.0%) previously underwent allogeneic hematopoietic stem cell transplantation (allo-HSCT) (Table 1).

**Table 1:** Patient characteristics

Characteristic		Patients (%)	
Total		288	
Sex	Male	191	(66.3)
	Female	97	(33.7)
Age (years)	< 75	220	(76.4)
	≥ 75	68	(23.6)
	Median (range)	64.0 (15–88)	
ECOG PS	0	105	(36.5)
	1	129	(44.8)
	≥ 2	54	(18.8)
Time from diagnosis to start of nivolumab treatment (years)	≤ 1	46	(16.0)
	> 1 to ≤ 3	118	(41.0)
	> 3	115	(39.9)
	Unknown	9	(3.1)
Ann Arbor Classification at start of nivolumab treatment	Stage I	8	(2.8)
	Stage II	59	(20.5)
	Stage III	89	(30.9)
	Stage IV	123	(42.7)
	Unknown	9	(3.1)
Prior allo-HSCT	No	265	(92.0)
	Yes	23	(8.0)
Prior liver disease	No	258	(89.6)
	Yes	30	(10.4)
Prior renal disease	No	268	(93.1)
	Yes	20	(6.9)
Prior lung disease	No	233	(80.9)
	Yes	55	(19.1)
Prior ILD	No	254	(88.2)
	Yes	34	(11.8)
Prior pulmonary emphysema or COPD	No	278	(96.5)
	Yes	10	(3.5)
Prior thyroid gland disorders	No	268	(93.1)
	Yes	20	(6.9)
Treatment line	1st	1	(0.3)
	2nd	27	(9.4)
	≥ 3rd	260	(90.3)

Values are *n* (%) of patients unless otherwise specified

*allo-HSCT* allogeneic hematopoietic stem cell transplantation, *COPD* chronic obstructive pulmonary disease, *ECOG PS* Eastern Cooperative Oncology Group performance status, *ILD* interstitial lung disease

### Treatment

The median number of nivolumab doses was 12.0 (range 1–28) (ESM Table 1). One hundred and two patients (35.4%) were continuing nivolumab at 12 months. Reasons for stopping nivolumab in the other 186 patients (64.6%) included AEs (23.3%), disease progression (22.9%), and lack of effectiveness (12.5%).

### Safety

TRAEs were reported in 183 patients (63.5%), including grade ≥ 3 TRAEs in 86 (29.9%) (Table 2, ESM Table 2). The most common TRAEs were infusion reaction (14.6%), hepatic functional abnormal (5.9%), ILD (5.6%), and hypothyroidism (5.2%). The incidence of TRAEs by SOC in this PMS was generally similar to the combined incidences in the two clinical trials (ESM Fig. 1) [8–11]. TRAEs by SOC that occurred at a higher frequency (by ≥ 2%) in this PMS than in those clinical trials were immune system disorders, cardiac disorders, hepatobiliary disorders, and renal and urinary disorders.

**Table 2:** TRAEs in ≥ 2% of patients in this PMS and in two clinical trials

TRAE by SOC/PT	This PMS (*n* = 288)		Clinical trials (*n* = 97)^a^	
Any TRAE	183	(63.5)	89	(91.8)
Infections and infestations	30	(10.4)	16	(16.5)
Herpes zoster	6	(2.1)	0	–
Pneumonia	9	(3.1)	3	(3.1)
Endocrine disorders	26	(9.0)	15	(15.5)
Hypothyroidism	15	(5.2)	10	(10.3)
Nervous system disorders	31	(10.8)	19	(19.6)
Peripheral neuropathy	7	(2.4)	4	(4.1)
Respiratory, thoracic and mediastinal disorders	28	(9.7)	17	(17.5)
Interstitial lung disease	16	(5.6)	1	(1.0)
Hepatobiliary disorders	25	(8.7)	2	(2.1)
Hepatic dysfunction	17	(5.9)	0	–
Skin and subcutaneous tissue disorders	31	(10.8)	32	(33.0)
Rash	11	(3.8)	18	(18.6)
Renal and urinary disorders	12	(4.2)	0	–
Renal disorder	7	(2.4)	0	–
General disorders and administration site conditions	25	(8.7)	44	(45.4)
Malaise	9	(3.1)	3	(3.1)
Fever	12	(4.2)	18	(18.6)
Investigations	36	(12.5)	29	(29.9)
Thrombocytopenia	6	(2.1)	2	(2.1)
Injury, poisoning, and procedural complications	42	(14.6)	17	(17.5)
Infusion reaction	42	(14.6)	17	(17.5)

Values are *n* (%) of patients

*PMS* post-marketing surveillance, *PT* preferred term, *SOC* system organ class, *TRAE* treatment-related adverse event

^a^Data were combined from two clinical trials: CheckMate 205 [8, 9] and ONO-4538-15[10, 11]

Possible relationships between death and nivolumab treatment could not be ruled out in eight patients. The TRAEs with an outcome of death were ILD (*n* = 2), death (*n* = 2), pseudomonal sepsis (*n* = 1), pulmonary edema (*n* = 1), pneumonia (*n* = 1), hemophagocytic lymphohistiocytosis (*n* = 1), and graft-versus-host disease (GVHD; *n* = 1); ILD and pseudomonal sepsis were both reported in one patient.

### Safety in subgroups of patients

The incidence of TRAEs differed significantly among some subgroups of patients divided by age, prior renal disease, and the time from cHL diagnosis to initiation of nivolumab (ESM Table 3).

Patients with an ECOG PS ≥ 2 or prior allo-HSCT were ineligible for the clinical trials. The incidence of any grade and grade ≥ 3 TRAEs in patients with an ECOG PS ≥ 2 were 61.1 and 37.0%, respectively, and were similar to the values in patients with an ECOG PS ≤ 1 (64.1 and 28.2%, respectively). The incidence of any grade and grade ≥ 3 TRAEs in patients with prior allo-HSCT were 82.6 and 52.2%, respectively, exceeding the values in patients without prior allo-HSCT (61.9 and 27.9%, respectively); the difference was statistically significant for grade ≥ 3 TRAEs (*P* = 0.0297).

### TRAEs of special interest

TRAEs of special interest that occurred in ≥ 5% of patients were infusion reactions (15.6%), hepatic failure/hepatic dysfunction/hepatitis/cholangitis sclerosing (13.2%), thyroid dysfunction (9.7%), and ILD (7.3%) (Table 3).

**Table 3:** TRAEs of special interest in this PMS and in two clinical trials

TRAE	This PMS (*n* = 288)		CheckMate 205 (*n* = 80)^a^		ONO-4538–15 (*n* = 17)^b^	
	Any grade	Grade 3–5	Any grade	Grade 3–5	Any grade	Grade 3–5
Interstitial lung disease	21 (7.3)	9 (3.1)	3 (3.8)	0	1 (5.9)	1 (5.9)
Myasthenia gravis, myocarditis, myositis, rhabdomyolysis	5 (1.7)	4 (1.4)	0	0	0	0
Colitis, enteritis, severe diarrhea	8 (2.8)	0	10 (12.5)	0	2 (11.8)	0
Type 1 diabetes mellitus	4 (1.4)	4 (1.4)	0	0	0	0
Hepatic failure, hepatic dysfunction, hepatitis, cholangitis sclerosing	38 (13.2)	22 (7.6)	11 (13.8)	4 (5.0)	1 (5.9)	0
Thyroid dysfunction	28 (9.7)	1 (0.3)	13 (16.3)	0	3 (17.6)	0
Pituitary disorder	7 (2.4)	1 (0.3)	1 (1.3)	0	0	0
Neurological disorder	10 (3.5)	2 (0.7)	5 (6.3)	0	1 (5.9)	0
Renal disorder	5 (1.7)	2 (0.7)	2 (2.5)	0	0	0
Adrenal disorder	2 (0.7)	0	0	0	0	0
Encephalitis	5 (1.7)	3 (1.0)	0	0	0	0
Severe skin disorder	3 (1.0)	2 (0.7)	0	0	0	0
Venous thromboembolism	0	0	1 (1.3)	0	0	0
Infusion reaction (within 24 h)	45 (15.6)	4 (1.4)	n.d	n.d	n.d	n.d
Infusion reaction	57 (19.8)	7 (2.4)	38 (47.5)	2 (2.5)	7 (41.2)	0
Serious blood disorder	7 (2.4)	7 (2.4)	0	0	0	0
Hemophagocytic syndrome	2 (0.7)	2 (0.7)	0	0	0	0
Tuberculosis	1 (0.3)	0	0	0	0	0
Cardiac disorders such as atrial fibrillation, bradycardia, and ventricular extrasystoles	7 (2.4)	4 (1.4)	0	0	0	0

Values are *n* (%) of patients

*PMS* post-marketing surveillance, *TRAE* treatment-related adverse event

^a^CheckMate 205 [8, 9]

^b^ONO-4538-15 [10, 11]

Multivariable analyses revealed that prior allo-HSCT for cHL was a risk factor for hepatic failure/hepatic dysfunction/hepatitis/cholangitis sclerosing (HR 2.80–4.30, ESM Table 4) and prior thyroid gland disorders was a risk factor for thyroid dysfunction (HR 2.97–3.48, ESM Table 5). The multivariable analyses failed to identify any independent risk factor for ILD (data not shown); however, the univariable analyses suggested trends toward a greater incidence of ILD in some subgroups of patients (e.g., patients with prior ILD, patients with abnormal finding on chest imaging [CT], or past/recent use of bleomycin; ESM Table 6). The percentage of patients with a history of past or recent use of bleomycin was 84.0 and 5.2%, respectively. However, the frequency of TRAEs did not differ between patients with or without history of bleomycin use (ESM Table 3).

Most of the TRAEs of special interest recovered/were recovering with appropriate treatments, such as corticosteroid, corticosteroid + immunosuppressant, or hormone replacement therapy (Table 4). Among patients with prior allo-HSCT, 75.0% of patients (six of eight patients) with hepatic failure/hepatic function disorder/hepatitis/cholangitis sclerosing recovered/were recovering (ESM Table 7). However, ≤ 50% of patients with hemophagocytic syndrome, type 1 diabetes mellitus, neurological disorder, or adrenal disorder recovered/were recovering.

**Table 4:** Treatments and outcomes of TRAEs of special interest

TRAE	Recovered/recovering		Non-recovery		Death		Unknown	
Interstitial lung disease (*n* = 21)	15	(71.4)	4	(19.0)	2	(9.5)	0	–
No treatment	2	(66.7)	1	(33.3)	0	–	0	–
Treated with a corticosteroid	13	(76.5)	2	(11.8)	2	(11.8)	0	–
Other treatment	0	–	1	(100)	0	–	0	–
Hepatic failure, hepatic dysfunction, hepatitis, cholangitis sclerosing (*n* = 38)	32	(84.2)	5	(13.2)	0	–	1	(2.6)
No treatment	16	(84.2)	2	(10.5)	0	–	1	(5.3)
Treated with a corticosteroid	10	(100)	0	–	0	–	0	–
Treated with a corticosteroid + immunosuppressant	1	(33.3)	2	(66.7)	0	–	0	–
Other treatment	4	(80.0)	1	(20.0)	0	–	0	–
Unknown	1	(100)	0	–	0	–	0	–
Thyroid dysfunction (*n* = 28)	18	(64.3)	9	(32.1)	0	–	1	(3.6)
No treatment	6	(75.0)	2	(25.0)	0	–	0	–
Hormone replacement therapy	10	(55.6)	7	(38.9)	0	–	1	(5.6)
Other treated	2	(100)	0	–	0	–	0	–
Pituitary disorder (*n* = 7)	5	(71.4)	1	(14.3)	0	–	1	(14.3)
No treatment	2	(66.7)	1	(33.3)	0	–	0	–
Treated with corticosteroid	2	(66.7)	0	-	0	–	1	(33.3)
Other treatment	1	(100)	0	-	0	–	0	–
Myasthenia gravis, myocarditis, myositis, rhabdomyolysis (*n* = 5)	4	(80.0)	1	(20.0)	0	–	0	–
Colitis, enteritis, severe diarrhea (*n* = 8)	8	(100)	0	–	0	–	0	–
Type 1 diabetes mellitus (*n* = 4)	2	(50.0)	2	(50.0)	0	–	0	–
Treated with insulin	2	(50.0)	2	(50.0)	0	–	0	–
Neurological disorder (*n* = 10)	5	(50.0)	5	(50.0)	0	–	0	–
No treatment	4	(57.1)	3	(42.9)	0	–	0	–
Treated with drugs for neurological disorder	1	(33.3)	2	(66.7)	0	–	0	–
Renal disorder (*n* = 5)	3	(60.0)	2	(40.0)	0	–	0	–
Adrenal disorder (*n* = 2)	1	(50.0)	1	(50.0)	0	–	0	–
Treated with a corticosteroid	1	(50.0)	1	(50.0)	0	–	0	–
Encephalitis (*n* = 5)	5	(100)	0	–	0	–	0	–
Severe skin disorder (*n* = 3)	3	(100)	0	–	0	–	0	–
Infusion reaction (within 24 h) (*n* = 45)	44	(97.8)	1	(2.2)	0	–	0	–
Serious blood disorder (*n* = 7)	6	(85.7)	1	(14.3)	0	–	0	–
Hemophagocytic syndrome (*n* = 2)	0	–	1	(50.0)	1	(50.0)	0	–
Tuberculosis (*n* = 1)	1	(100)	0	–	0	–	0	–
Cardiac disorders such as atrial fibrillation, bradycardia, ventricular extrasystole) (*n* = 7)	5	(71.4)	2	(28.6)	0	–	0	–

Values are *n* (%) of patients

*TRAE* treatment-related adverse event

### GVHD

Among 23 patients with prior allo-HSCT, six developed GVHD (ESM Table 8). All six patients were treated with steroids; three were recovering, two had not recovered, and one patient died. Other than these cases, GVHD was observed in two patients who underwent allo-HSCT after receiving nivolumab (grade 4 in one patient and grade 2 in one patient). Both patients were treated with steroids and recovered/were recovering.

### Effectiveness

The best responses were CR in 63 patients (22.3%), PR in 111 (39.4%), SD in 59 (20.9%), and PD in 35 (12.4%). The response could not be determined in 14 patients. The ORR was 61.7% (95% confidence interval [CI] 55.8–67.4%). Of 282 patients assessed for survival, 237 (84.0%) were alive at 6 months and 207 (73.4%) were alive at 12 months.

## Discussion

Following the approval of nivolumab for r/r cHL in Japan, this PMS was conducted to obtain data on its safety and effectiveness in the real-world setting. Unlike prior clinical trials, all patients treated with nivolumab for cHL were registered without any specific exclusion criteria. Therefore, the patient population may more closely represent the patients who suffer from cHL and are treated with nivolumab in Japan than in clinical trials. Notably, the overall incidence of TRAEs (63.5%) was not higher than that observed in ONO-4538-15 and CheckMate 205 combined (91.8%) (Table 2). The incidence of grade ≥ 3 TRAEs (29.9%, ESM Table 2) was also similar to that in both trials (ONO-4538-15: 23.5%; CheckMate 205: 25.0%). However, observational studies (including PMS) may underestimate the incidence of TRAEs compared with clinical trials.

The clinical trials excluded patients with prior allo-HSCT. In the present analyses, prior allo-HSCT was found to be a risk factor for hepatic failure/hepatic dysfunction/hepatitis/cholangitis sclerosing (HR 2.80–4.30, ESM Table 4). Liver-related TRAEs are a common symptom of GVHD, which is a potential complication of allo-HSCT. Liver-related TRAEs were observed in eight patients with prior allo-HSCT, and GVHD was reported as a TRAE in two of those patients. This suggests that liver-related TRAEs in patients with prior allo-HSCT may be associated with GVHD. In addition, another study reported a higher incidence rate of abnormal hepatic function in patients with prior allo-HSCT than in patients without prior allo-HSCT (20 vs 7%) [15]. Therefore, the higher incidence of liver-related TRAEs than in the previous clinical trials is partially responsible for the higher incidence of TRAEs in patients with prior allo-HSCT. Liver-related TRAEs were reported in nivolumab-treated patients with other carcinomas and are not new concerns [16–19]. We also noted that more than 80% of patients recovered/were recovering with appropriate treatment (Table 4). Regarding hepatic failure/hepatic dysfunction/hepatitis/cholangitis sclerosing, the proportion of patients that recovered/were recovering was similar between patients with and without prior allo-HSCT (75.0 vs 86.7%, respectively, ESM Table 7).

Immune system disorders (4.2%) were more frequent in this PMS than in CheckMate 205 (2.5%) and ONO-4538-15 (0%) (ESM Fig. 1). Immune system disorders included TRAEs related to transplantation complications (acute/chronic GVHD in eight patients and engraftment syndrome in one patient). The exclusion of patients with prior allo-HSCT from the clinical trials may contribute to the higher incidence of immune system disorders in this PMS. Prior reports described an increased frequency of GVHD with administration of an anti-PD-1 monoclonal antibody (mAb) administration before and after allo-HSCT [9, 20–26]. Since detailed information about GVHD is not available, it is difficult to directly compare the prevalence of GVHD in this study with that reported in other studies. It should be noted that GVHD occurred in six of 23 patients with prior allo-HSCT (26.1%), and half of those patients did not recover or died. Considering earlier reports and our data, physicians should pay careful attention to GVHD when administering nivolumab before and after allo-HSCT.

Several TRAEs (renal and urinary disorders, cardiac disorders, and herpes zoster) were more frequent in this PMS than in the prior clinical trial. However, the sample sizes of CheckMate 205 (*n* = 80) and ONO-4538-15 (*n* = 17) were too small to detect these TRAEs. Nevertheless, these are not new safety signals for nivolumab having been reported previously [16, 18].

ILD, a TRAE of special interest, was more frequent in this PMS (7.3%) than in CheckMate 205 (3.8%). Pneumonitis and other lung disorders are well recognized among patients treated with immune checkpoint inhibitors [27]. Furthermore, Japanese individuals tend to develop ILD more frequently (4 vs 0.2% for the rest of the world) [28, 29], possibly due to genetic factors [30], which may explain the higher frequency in this PMS. Prior ILD is a potential risk factor for ILD in patients treated with immune checkpoint inhibitors [30]. We found that ILD was numerically, but not significantly, more frequent in patients with prior ILD than in patients without prior ILD (14.7 vs 6.3%, HR 2.48, 95% CI 0.90–6.83; ESM Table 6). Patients with any abnormal chest radiographic findings had a numerically, but not significantly, higher incidence of ILD than patients with no abnormal chest findings (10.9% vs 4.3%, HR 2.54, 95% CI 0.92–7.00). Prior ILD and presence of any chest radiographic abnormalities were associated with the risk of ILD in earlier PMS of patients with lung cancer and melanoma [18, 19]. Therefore, the higher incidence of ILD in this PMS than in the clinical trials may be attributable to genetic factors and/or the inclusion of more patients with risk factors for ILD (e.g., prior ILD). Further data are needed to better understand this association.

Several other TRAEs of special interest were reported here but not in CheckMate 205 or ONO-4538-15 (Table 3). However, these TRAEs of special interest were reported in prior PMS of nivolumab [16–19] and do not represent new safety signals.

Finally, regarding the effectiveness of nivolumab, the ORR was 61.7% (95% CI 55.8–67.4%), similar to that in CheckMate 205 (66.3%, 95% CI 54.8–76.4%) [8], but numerically lower than that in ONO-4538-15 (81.3%, 95% CI 54.4–96.0%) [10]. A possible limitation of this outcome is that the responses were not centrally assessed. If we consider the overlapping 95% CIs and the limited number of patients in the Japanese phase II trial, the ORR in this PMS is within the range reported in the clinical trials.

The results of this PMS should be interpreted cautiously because of its potential limitations, including the lack of a control group, the relatively short (1 year) follow-up, and lack of central review for safety and effectiveness assessment. Due to the lack of detailed information on GVHD (such as the new onset or worsening of preexisting GVHD, the severity/grade of prior GVHD, the conditioning regimen, the donor’s or recipient’s status, or the presence of prophylaxis), it is difficult to directly compare its prevalence in this PMS with that reported in other studies.

In conclusion, the safety profile of nivolumab in this PMS of Japanese patients with r/r cHL was similar to that reported in earlier clinical trials of r/r cHL and PMS for other malignancies. We identified no new safety concerns, based on TRAEs of special interest, that were not previously reported in clinical trials and/or PMS for other malignancies in Japan. The effectiveness of nivolumab treatment in patients with r/r cHL in this real-world setting was comparable with that reported in the earlier clinical trials. Overall, these results demonstrate that nivolumab is a safe and effective treatment for patients with r/r cHL in the real-world setting in Japan.

### Supplementary Information

Below is the link to the electronic supplementary material.

Supplementary file1 (PDF 364 KB)

## Data Availability

Qualified researchers may request Ono Pharmaceutical Co., Ltd. to disclose individual patient-level data from clinical studies through the following website: https://www.clinicalstudydatarequest.com/. For more information on Ono Pharmaceutical Co., Ltd.’s Policy for the Disclosure of Clinical Study Data, please see the following website: https://www.ono.co.jp/eng/rd/policy.html.
