# Supplementary material for: Post-marketing surveillance of the safety and effectiveness of nivolumab for classic Hodgkin lymphoma in Japan
Source: Int J Hematol. 2024 Mar 23;119(6):667–76. doi: 10.1007/s12185-024-03734-y (PMC11136857; doi:10.1007/s12185-024-03734-y)
Supplement: Supplementary file 1 — Supplementary file1 (PDF 364 KB) [file 12185_2024_3734_MOESM1_ESM.pdf]

**Electronic supplementary materials**


| Content | Page |
|---|---|
| **ESM Table 1** Administration of nivolumab | 2 |
| **ESM Table 2** TRAEs in ≥2% of patients by grade | 3 |
| **ESM Table 3** Subgroup analysis of TRAEs | 4 |
| **ESM Table 4** Risk factors for hepatic failure/hepatic dysfunction/hepatitis/cholangitis sclerosing (multivariable analysis; Fine and Gray proportional subdistribution hazards model) | 7 |
| **ESM Table 5.** Risk factors for thyroid dysfunction (multivariable analysis; Fine and Gray proportional subdistribution hazards model) | 9 |
| **ESM Table 6** Risk factors for ILD (univariable analysis) | 10 |
| **ESM Table 7** Grade and outcome of hepatic failure/hepatic dysfunction/hepatitis/cholangitis sclerosing according to prior allo-HSCT | 12 |
| **ESM Table 8** Cases of graft-versus-host disease reported during treatment with nivolumab | 13 |
| **ESM Figure 1** Adverse drug reactions, by system organ class, in this post-marketing surveillance and in previous clinical trials (CheckMate 205 [1,2] and ONO-4538-15 [3,4]) | 14 |
| **References** | 15 |

**ESM Table 1** Administration of nivolumab

| Item | Category | Patients, *n* (%) | |
|---|---|---|---|
| Number of doses | 1–4 | 65 | (22.6) |
| | 5–8 | 46 | (16.0) |
| | 9–12 | 36 | (12.5) |
| | 13–16 | 32 | (11.1) |
| | 17–20 | 25 | (8.7) |
| | 21–24 | 34 | (11.8) |
| | ≥25 | 50 | (17.4) |
| | Median (range) | 12.0 (1–28) | |
| Dose (mg) | ≤100 | 7 | (2.4) |
| | >100 to ≤200 | 147 | (51.0) |
| | >200 to ≤300 | 131 | (45.5) |
| | >300 | 1 | (0.3) |
| | Unknown | 2 | (0.7) |
| | Mean ± SD | 189.7 ± 48.5 (*n* = 286) | |
| | Median (range) | 190.4 (27.0–312.0 (*n* = 288) | |
| Dose category | Always 3 mg/kg | 144 | (50.0) |
| | Always 240 mg/body | 84 | (29.2) |
| | Switched to 240 mg/body | 26 | (9.0) |
| | Other | 33 | (11.5) |
| | Unknown | 1 | (0.3) |

*SD* standard deviation

**ESM Table 2** TRAEs in ≥2% of patients by grade

| TRAE | Total, *n* (%) | | Grade 3–5, *n* (%) | |
|---|---|---|---|---|
| Any TRAE | 183 | (63.5) | 86 | (29.9) |
| Infections and infestations | 30 | (10.4) | 14 | (4.9) |
| Herpes zoster | 6 | (2.1) | 3 | (1.0) |
| Pneumonia | 9 | (3.1) | 5 | (1.7) |
| Endocrine disorders | 26 | (9.0) | 2 | (0.7) |
| Hypothyroidism | 15 | (5.2) | 0 | - |
| Nervous system disorders | 31 | (10.8) | 11 | (3.8) |
| Peripheral neuropathy | 7 | (2.4) | 1 | (0.3) |
| Respiratory, thoracic, and mediastinal disorders | 28 | (9.7) | 12 | (4.2) |
| Interstitial lung disease | 16 | (5.6) | 7 | (2.4) |
| Hepatobiliary disorders | 25 | (8.7) | 19 | (6.6) |
| Hepatic dysfunction | 17 | (5.9) | 14 | (4.9) |
| Skin and subcutaneous tissue disorders | 31 | (10.8) | 7 | (2.4) |
| Rash | 11 | (3.8) | 1 | (0.3) |
| Renal and urinary disorders | 12 | (4.2) | 4 | (1.4) |
| Renal disorder | 7 | (2.4) | 2 | (0.7) |
| General disorders and administration site conditions | 25 | (8.7) | 6 | (2.1) |
| Malaise | 9 | (3.1) | 1 | (0.3) |
| Fever | 12 | (4.2) | 2 | (0.7) |
| Investigations | 36 | (12.5) | 11 | (3.8) |
| Thrombocytopenia | 6 | (2.1) | 4 | (1.4) |
| Injury, poisoning, and procedural complications | 42 | (14.6) | 4 | (1.4) |
| Infusion reaction | 42 | (14.6) | 4 | (1.4) |

*PMS* post-marketing surveillance, *TRAE* treatment-related adverse event

**ESM Table 3** Subgroup analysis of TRAEs

| Characteristic | | Patients, *N* | (%) | Patients with any ADR, *n* | Incidence rate (%) | 95% CI for the incidence rate | *P*-value[a] |
|---|---|---|---|---|---|---|---|
| Overall | | 288 | | 183 | (63.5) | [57.7–69.1] | |
| Sex | Male | 191 | (66.3) | 121 | (63.4) | [56.1–70.2] | 1.0000[F] |
| | Female | 97 | (33.7) | 62 | (63.9) | [53.5–73.4] | |
| Age (years) | <75 | 220 | (76.4) | 148 | (67.3) | [60.6–73.4] | 0.0182[W] |
| | ≥75 | 68 | (23.6) | 35 | (51.5) | [39.0–63.8] | |
| ECOG PS | 0–1 | 234 | (81.3) | 150 | (64.1) | [57.6–70.2] | 0.6819[W] |
| | 2–4 | 54 | (18.8) | 33 | (61.1) | [46.9–74.1] | |
| Medical history | No | 81 | (28.1) | 44 | (54.3) | [42.9–65.4] | 0.0562[F] |
| | Yes | 207 | (71.9) | 139 | (67.1) | [60.3–73.5] | |
| Prior liver disease | No | 258 | (89.6) | 160 | (62.0) | [55.8–68.0] | 0.1598[F] |
| | Yes | 30 | (10.4) | 23 | (76.7) | [57.7–90.1] | |
| Prior renal disease | No | 268 | (93.1) | 165 | (61.6) | [55.5–67.4] | 0.0139[F] |
| | Yes | 20 | (6.9) | 18 | (90.0) | [68.3–98.8] | |
| Prior lung disease | No | 233 | (80.9) | 149 | (63.9) | [57.4–70.1] | 0.7581[F] |
| | Yes | 55 | (19.1) | 34 | (61.8) | [47.7–74.6] | |
| Prior thyroid gland disorders | No | 268 | (93.1) | 169 | (63.1) | [57.0–68.9] | 0.6347[F] |
| | Yes | 20 | (6.9) | 14 | (70.0) | [45.7–88.1] | |
| Prior autoimmune disease | No | 264 | (91.7) | 169 | (64.0) | [57.9–69.8] | 0.6590[F] |
| | Yes | 24 | (8.3) | 14 | (58.3) | [36.6–77.9] | |
| Prior ILD | No | 254 | (88.2) | 162 | (63.8) | [57.5–69.7] | 0.8507[F] |
| | Yes | 34 | (11.8) | 21 | (61.8) | [43.6–77.8] | |
| Prior pulmonary emphysema or COPD | No | 278 | (96.5) | 176 | (63.3) | [57.3–69.0] | 0.7515[F] |
| | Yes | 10 | (3.5) | 7 | (70.0) | [34.8–93.3] | |
| Prior pulmonary infection | No | 275 | (95.5) | 174 | (63.3) | [57.3–69.0] | 0.7745[F] |
| | Yes | 13 | (4.5) | 9 | (69.2) | [38.6–90.9] | |

| **Characteristic** | | **Patients, *N*** | **(%)** | **Patients with any ADR, *n*** | **Incidence rate (%)** | **95% CI for the incidence rate** | ***P*-value[a]** |
|---|---|---|---|---|---|---|---|
| Ann Arbor classification at start of nivolumab treatment | Stage I | 8 | (2.8) | 4 | (50.0) | [15.7–84.3] | 0.1702[W] |
| | Stage II | 59 | (20.5) | 32 | (54.2) | [40.8–67.3] | |
| | Stage III | 89 | (30.9) | 60 | (67.4) | [56.7–77.0] | |
| | Stage IV | 123 | (42.7) | 81 | (65.9) | [56.8–74.2] | |
| | Unknown | 9 | (3.1) | 6 | (66.7) | - | |
| Abnormal chest imaging findings (radiography), (*N* = 257) | No | 190 | (73.9) | 115 | (60.5) | [53.2–67.5] | 0.0719[F] |
| | Yes | 64 | (24.9) | 47 | (73.4) | [60.9–83.7] | |
| | Unknown | 3 | (1.2) | 3 | (100) | - | |
| Abnormal chest imaging findings (CT), (*N* = 254) | No | 116 | (45.7) | 66 | (56.9) | [47.4–66.1] | 0.0514[F] |
| | Yes | 138 | (54.3) | 95 | (68.8) | [60.4–76.4] | |
| Abnormal chest imaging findings (MRI), (*N* = 4) | No | 2 | (50.0) | 1 | (50.0) | [1.3–98.7] | 1.0000[F] |
| | Yes | 2 | (50.0) | 2 | (100) | [15.8–100.0] | |
| Prior chest radiation | No | 245 | (85.1) | 154 | (62.9) | [56.5–68.9] | 0.7299[F] |
| | Yes | 42 | (14.6) | 28 | (66.7) | [50.5–80.4] | |
| | Unknown | 1 | (0.3) | 1 | (100) | - | |
| Prior autologous HSCT for cHL | No | 212 | (73.6) | 134 | (63.2) | [56.3–69.7] | 0.8901[F] |
| | Yes | 76 | (26.4) | 49 | (64.5) | [52.7–75.1] | |
| Prior allogeneic HSCT for cHL | No | 265 | (92.0) | 164 | (61.9) | [55.7–67.8] | 0.0687[F] |
| | Yes | 23 | (8.0) | 19 | (82.6) | [61.2–95.0] | |
| Prior radiotherapy for cHL | No | 200 | (69.4) | 122 | (61.0) | [53.9–67.8] | 0.1809[F] |
| | Yes | 86 | (29.9) | 60 | (69.8) | [58.9–79.2] | |
| | Unknown | 2 | (0.7) | 1 | (50.0) | - | |
| Treatment line | First | 1 | (0.3) | 1 | (100) | [2.5–100.0] | 0.0508[W] |
| | Second | 27 | (9.4) | 12 | (44.4) | [25.5–64.7] | |
| | Third/later | 260 | (90.3) | 170 | (65.4) | [59.3–71.2] | |
| Past use of bleomycin[b] | No | 46 | (16.0) | 30 | (65.2) | [49.8–78.6] | 0.8682[F] |
| | Yes | 242 | (84.0) | 153 | (63.2) | [56.8–69.3] | |
| Recent use of bleomycin[c] | No | 273 | (94.8) | 175 | (64.1) | [58.1–69.8] | 0.4183[F] |
| | Yes | 15 | (5.2) | 8 | (53.3) | [26.6–78.7] | |

| Characteristic | | Patients, *N* | (%) | Patients with any ADR, *n* | Incidence rate (%) | 95% CI for the incidence rate | *P*-value[a] |
|---|---|---|---|---|---|---|---|
| Time from diagnosis of cHL to start of nivolumab treatment (years) | ≤1 | 46 | (16.0) | 19 | (41.3) | [27.0–56.8] | 0.0151[W] |
| | >1 to ≤3 | 118 | (41.0) | 79 | (66.9) | [57.7–75.3] | |
| | >3 | 115 | (39.9) | 78 | (67.8) | [58.5–76.2] | |
| | Unknown | 9 | (3.1) | 7 | (77.8) | - | |
| Number of doses | 1–4 | 65 | (22.6) | 43 | (66.2) | [53.4–77.4] | 0.1003[W] |
| | 5–8 | 46 | (16.0) | 30 | (65.2) | [49.8–78.6] | |
| | 9–12 | 36 | (12.5) | 25 | (69.4) | [51.9–83.7] | |
| | 13–16 | 32 | (11.1) | 22 | (68.8) | [50.0–83.9] | |
| | 17–20 | 25 | (8.7) | 18 | (72.0) | [50.6–87.9] | |
| | 21–24 | 34 | (11.8) | 21 | (61.8) | [43.6–77.8] | |
| | ≥25 | 50 | (17.4) | 24 | (48.0) | [33.7–62.6] | |

[a] Fisher's exact test (F) or Wilcoxon's rank-sum test (W)

[b] Any historical treatment with the indicated drug

[c] Indicated drug used as the most recent therapy before starting nivolumab

*ADR* adverse drug reaction, *cHL* classic Hodgkin's lymphoma, *COPD* chronic obstructive pulmonary disease, *CT* computed tomography, *ECOG PS* Eastern Cooperative Oncology Group performance status, *HSCT* hematopoietic stem cell transplantation, *ILD* interstitial lung disease, *MRI* magnetic resonance imaging, *TRAE* treatment-related adverse event

**ESM Table 4** Risk factors for hepatic failure/hepatic dysfunction/hepatitis/cholangitis sclerosing (multivariable analysis; Fine and Gray proportional subdistribution hazards model)

| **Explanatory variables** | **Comparison** | **HR** | **(95% CI)** |
|---|---|---|---|
| **Model 1** | | | |
| Prior allo-HSCT for cHL | Yes vs No | 3.09 | (1.38–6.92) |
| Sex | Male vs Female | 1.27 | (0.63–2.53) |
| **Model 2** | | | |
| Prior allo-HSCT for cHL | Yes vs No | 2.80 | (1.23–6.36) |
| Age group (years) | ≥75 vs <75 | 0.68 | (0.28–1.64) |
| **Model 3** | | | |
| Prior allo-HSCT for cHL | Yes vs No | 3.37 | (1.47–7.74) |
| ECOG PS | 2–4 vs 0–1 | 1.71 | (0.78–3.47) |
| **Model 4** | | | |
| Prior allo-HSCT for cHL | Yes vs No | 3.06 | (1.39–6.75) |
| Prior liver disease | Yes vs No | 1.75 | (0.75–4.08) |
| **Model 5** | | | |
| Prior allo-HSCT for cHL | Yes vs No | 2.97 | (1.33–6.65) |
| Prior autoimmune disease | Yes vs No | 0.70 | (0.17–2.89) |
| **Model 6** | | | |
| Prior allo-HSCT for cHL | Yes vs No | 3.67 | (1.58–8.51) |
| Ann Arbor stage at the start of nivolumab treatment | III–IV vs I–II | 0.51 | (0.25–1.04) |
| **Model 7** | | | |
| Prior allo-HSCT for cHL | Yes vs No | 4.30 | (1.39–13.32) |
| Prior HSCT for cHL | Yes vs No | 0.65 | (0.25–1.70) |
| **Model 8** | | | |
| Prior allo-HSCT for cHL | Yes vs No | 3.05 | (1.38–6.77] |
| Treatment line | 2nd vs ≥3rd | 0.87 | (0.26–2.96) |
| **Model 9** | | | |
| Prior allo-HSCT for cHL | Yes vs No | 3.36 | (1.46–7.76) |
| Past use of brentuximab[a] | Yes vs No | 0.43 | (0.16–1.19) |

| Explanatory variables | Comparison | HR | (95% CI) |
|---|---|---|---|
| **Model 10** | | | |
| Prior allo-HSCT for cHL | Yes vs No | 2.88 | (1.30–6.38) |
| Recent use of brentuximab[b] | Yes vs No | 0.64 | (0.34–1.20) |
| **Model 11** | | | |
| Prior allo-HSCT for cHL | Yes vs No | 2.96 | (1.32–6.64) |
| Past use of dacarbazine[a] | Yes vs No | 0.78 | (0.35–1.76) |
| **Model 12** | | | |
| Prior allo-HSCT for cHL | Yes vs No | 3.02 | (1.35–6.75) |
| Recent use of dacarbazine[b] | Yes vs No | 0.83 | (0.20–3.53) |
| **Model 13** | | | |
| Prior allo-HSCT for cHL | Yes vs No | 2.93 | (1.29–6.66) |
| Past use of doxorubicin[a] | Yes vs No | 0.59 | (0.24–1.45) |
| **Model 14** | | | |
| Prior allo-HSCT for cHL | Yes vs No | 2.99 | (1.35–6.64) |
| Recent use of doxorubicin[b] | Yes vs No | 0.56 | (0.14–2.30) |
| **Model 15** | | | |
| Prior allo-HSCT for cHL | Yes vs No | 3.17 | (1.40–7.22) |
| CRP before the start of nivolumab treatment (mg/dL) | ≥5 vs <5 | 1.58 | (0.82–3.08) |

[a] Any historical treatment with the indicated drug

[b] Indicated drug used as the most recent therapy before starting nivolumab

*allo-HSCT* allogeneic hematopoietic stem cell transplantation, *cHL* classic Hodgkin's lymphoma, *CI* confidence interval, *CRP* C-reactive protein, *ECOG PS* Eastern Cooperative Oncology Group performance status, *HR* hazards ratio, *HSCT* hematopoietic stem cell transplantation

**ESM Table 5.** Risk factors for thyroid dysfunction (multivariable analysis; Fine and Gray proportional subdistribution hazards model)

| Explanatory variables | Comparison | HR | (95% CI) |
|---|---|---|---|
| **Model 1** | | | |
| Prior thyroid gland disorders | Yes vs No | 2.97 | (1.08–8.19) |
| Sex | Male vs Female | 0.79 | (0.36–1.72) |
| **Model 2** | | | |
| Prior thyroid gland disorders | Yes vs No | 3.22 | (1.18–8.81) |
| Age group (years) | ≥75 vs <75 | 0.52 | (0.18–1.50) |
| **Model 3** | | | |
| Prior thyroid gland disorders | Yes vs No | 3.17 | (1.16–8.68) |
| ECOG PS | 2-4 vs 0-1 | 0.80 | (0.27–2.40) |
| **Model 4** | | | |
| Prior thyroid gland disorders | Yes vs No | 3.24 | (1.21–8.69) |
| Prior autoimmune disease | Yes vs No | 0.84 | (0.20–3.47) |
| **Model 5** | | | |
| Prior thyroid gland disorders | Yes vs No | 3.22 | (1.24–8.36) |
| Ann Arbor stage at the start of nivolumab treatment | III–IV vs I–II | 0.76 | (0.34–1.68) |
| **Model 6** | | | |
| Prior thyroid gland disorders | Yes vs No | 3.48 | (1.29–9.39) |
| Prior allo-HSCT for cHL | Yes vs No | 0.34 | (0.05–2.60) |
| **Model 7** | | | |
| Prior thyroid gland disorders | Yes vs No | 3.26 | (1.22–8.72) |
| Treatment line | 2nd vs ≥3rd | 0.74 | (0.17–3.23) |

*allo-HSCT* allogeneic hematopoietic stem cell transplantation, *cHL* classic Hodgkin's lymphoma, *CI* confidence interval, *ECOG PS* Eastern Cooperative Oncology Group performance status, *HR* hazards ratio

**ESM Table 6** Risk factors for ILD (univariable analysis)

| Explanatory variables | Category | *N* | Patients with ILD, *n* (%) | Comparison between risk factor classifications (univariable analysis) | | |
|---|---|---|---|---|---|---|
| | | | | **Comparison** | **HR** | **(95% CI)** |
| Sex | Male | 191 | 15 (7.9) | Male vs Female | 1.27 | (0.49–3.30) |
| | Female | 97 | 6 (6.2) | | | |
| Age (years) | <75 | 220 | 14 (6.4) | ≥75 vs <75 | 1.61 | (0.65–3.99) |
| | ≥75 | 68 | 7 (10.3) | | | |
| ECOG PS | 0–1 | 234 | 14 (6.0) | 2–4 vs 0–1 | 2.22 | (0.90–5.46) |
| | 2–4 | 54 | 7 (13.0) | | | |
| Prior autoimmune disease | No | 264 | 20 (7.6) | Yes vs No | 0.53 | (0.07–3.84) |
| | Yes | 24 | 1 (4.2) | | | |
| Prior ILD | No | 254 | 16 (6.3) | Yes vs No | 2.48 | (0.90–6.83) |
| | Yes | 34 | 5 (14.7) | | | |
| Prior emphysema/COPD | No | 278 | 20 (7.2) | Yes vs No | 1.36 | (0.19–9.73) |
| | Yes | 10 | 1 (10.0) | | | |
| Prior lung infection | No | 275 | 21 (7.6) | Yes vs No | - | - |
| | Yes | 13 | 0 | | | |
| Ann Arbor stage at start of nivolumab treatment | I–II | 67 | 3 (4.5) | III–IV vs I–II | 1.92 | (0.56–6.54) |
| | III–IV | 212 | 18 (8.5) | | | |
| Abnormal finding on chest imaging (CT), *n* = 254 | No | 116 | 5 (4.3) | Yes vs No | 2.54 | (0.92–7.00) |
| | Yes | 138 | 15 (10.9) | | | |
| Prior thoracic irradiation | No | 245 | 18 (7.3) | Yes vs No | 0.65 | (0.15–2.84) |
| | Yes | 42 | 2 (4.8) | | | |
| Treatment line | 1st | 1 | 0 | 2nd vs ≥3rd | 1.06 | (0.25–4.54) |
| | 2nd | 27 | 2 (7.4) | | | |
| | ≥3rd | 260 | 19 (7.3) | | | |

| Explanatory variables | Category | *N* | Patients with ILD, *n* (%) | Comparison between risk factor classifications (univariable analysis) | | |
|---|---|---|---|---|---|---|
| | | | | **Comparison** | **HR** | **(95% CI)** |
| Past use of bleomycin[a] | No | 46 | 3 (6.5) | Yes vs No | 1.12 | (0.33–3.80) |
| | Yes | 242 | 18 (7.4) | | | |
| Recent use of bleomycin[b] | No | 273 | 21 (7.7) | Yes vs No | - | - |
| | Yes | 15 | 0 | | | |
| CRP before the start of nivolumab treatment (mg/dL) | <5 | 179 | 10 (5.6) | ≥5 vs <5 | 2.01 | (0.85–4.71) |
| | ≥5 | 99 | 11 (11.1) | | | |

[a] Any historical treatment with the indicated drug

[b] Indicated drug used as the most recent therapy before starting nivolumab

*CI* confidence interval, *COPD* chronic obstructive pulmonary disease, *CRP* C-reactive protein, *CT* computed tomography, *ECOG PS* Eastern Cooperative Oncology Group performance status, *HR* hazard ratio, *ILD* interstitial lung disease

**ESM Table 7** Grade and outcome of hepatic failure/hepatic dysfunction/hepatitis/cholangitis sclerosing according to prior allo-HSCT

| **Prior allo-HSCT** | **Grade** | | | | | | **Outcome** | | | | | | | |
|---|---|---|---|---|---|---|---|---|---|---|---|---|---|---|
| | **Any grade** | | **Grade 3–5** | | **Unknown** | | **Recovered/recovering** | | **Non-recovery** | | **Death** | | **Unknown** | |
| No (*N* = 265) | 30 | (11.3) | 16 | (6.0) | 0 | (0) | 26 | (86.7) | 3 | (10.0) | 0 | (0) | 1 | (3.3) |
| Yes (*N* = 23) | 8 | (34.8) | 6 | (26.1) | 1 | (4.3) | 6 | (75.0) | 2 | (25.0) | 0 | (0) | 0 | (0) |

Values are *n* (%)

*allo-HSCT* allogeneic hematopoietic stem cell transplantation

**ESM Table 8** Cases of graft-versus-host disease reported during treatment with nivolumab

| **Patient #** | **Sex** | **Age** | **PT** | **Grade** | **Time (days)[a]** | **Treatment for AE** | **Outcome** |
|---|---|---|---|---|---|---|---|
| 1 | Male | 45 | Graft-versus-host disease | 1 | 2 | Topical steroid[b] | Recovering |
| 2 | Male | 33 | Graft-versus-host disease | 2 | 21 | Prednisolone | Recovering |
| 3 | Female | 33 | Acute cutaneous graft-versus-host disease | 3 | 40 | Methylprednisolone, clobetasol propionate | Recovering |
| 4 | Male | 25 | Acute graft-versus-host disease | 3 | 87 | Prednisolone, anti-human thymocyte immunoglobulin, rabbit | Non-recovered |
| 5 | Female | 23 | Chronic graft-versus-host disease | 3 | 183 | Predonine | Non-recovered |
| 6 | Female | 15 | Graft-versus-host disease | 5 | 147 | Steroid[b] | Death |

[a] Time from start of nivolumab treatment to onset of AE

[b] Details are unknown

*AE* adverse event, *PT* preferred term

**ESM Figure 1** Adverse drug reactions, by system organ class, in this post-marketing surveillance and in previous clinical trials (CheckMate 205 [1,2] and ONO-4538-15 [3,4])
*ADR* adverse drug reaction

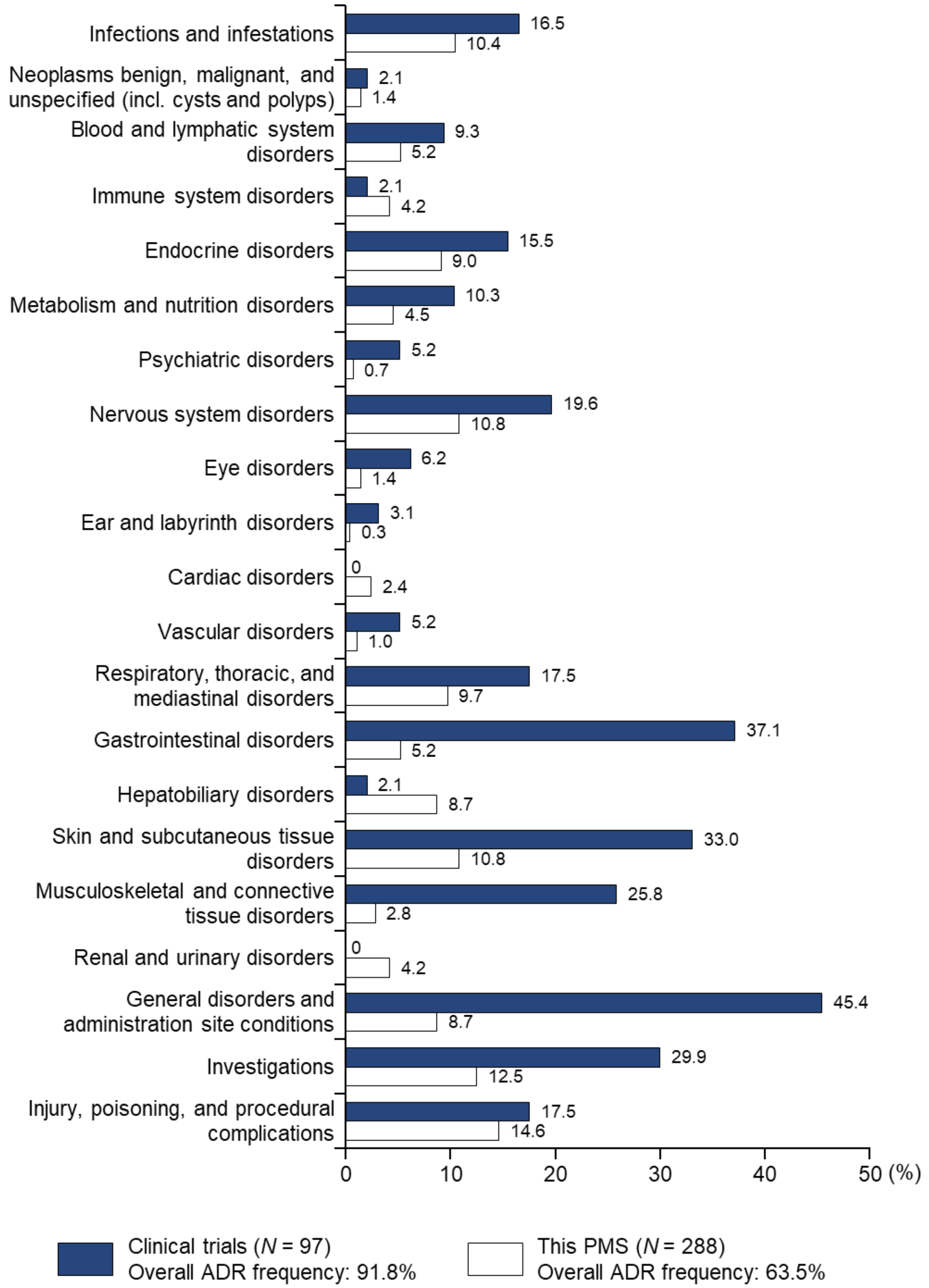

**References**

1. Younes A, Santoro A, Shipp M, Zinzani PL, Timmerman JM, Ansell S, et al. Nivolumab for classical Hodgkin's lymphoma after failure of both autologous stem-cell transplantation and brentuximab vedotin: a multicentre, multicohort, single-arm phase 2 trial. Lancet Oncol. 2016;17(9):1283–94. https://doi.org/10.1016/s1470-2045(16)30167-x.

2. Armand P, Engert A, Younes A, Fanale M, Santoro A, Zinzani PL, et al. Nivolumab for relapsed/refractory classic Hodgkin lymphoma after failure of autologous hematopoietic cell transplantation: extended follow-up of the multicohort single-arm phase II CheckMate 205 trial. J Clin Oncol. 2018;36(14):1428–39. https://doi.org/10.1200/jco.2017.76.0793.

3. Maruyama D, Hatake K, Kinoshita T, Fukuhara N, Choi I, Taniwaki M, et al. Multicenter phase II study of nivolumab in Japanese patients with relapsed or refractory classical Hodgkin lymphoma. Cancer Sci. 2017;108(5):1007–12. https://doi.org/10.1111/cas.13230.

4. Maruyama D, Terui Y, Yamamoto K, Fukuhara N, Choi I, Kuroda J, et al. Final results of a phase II study of nivolumab in Japanese patients with relapsed or refractory classical Hodgkin lymphoma. Jpn J Clin Oncol. 2020;50(11):1265–73. https://doi.org/10.1093/jjco/hyaa117.
